# Parietal complication of the hydatid disease: Comprehensive literature review

**DOI:** 10.1097/MD.0000000000010671

**Published:** 2018-05-25

**Authors:** Sami Akbulut

**Affiliations:** Department of Surgery and Liver Transplant Institute, Inonu University Faculty of Medicine, Malatya, Turkey.

**Keywords:** cutaneous involvement, cysto-cutaneous fistulization, hydatid disease, parietal complications

## Abstract

**Background:**

The aim of the study was to provide an overview of the medical literature on parietal complications of hydatid disease (HD).

**Methods:**

A literature search was conducted on PubMed, Medline, Google Scholar, and Google databases in accordance with the Preferred Reporting Items for Systematic Reviews and Meta-Analyses guidelines, using keywords to identify articles related to parietal complications of HD in the thoracic and abdominal cavities. The following keywords were used: HD, hydatid cyst, cystic echinococcosis, alveolar echinococcosis, abdominal HD, thoracic HD, parietal complication, cutaneous fistulization, cystocutaneous fistulization, cutaneous involvement, external rupture, external fistulization, subcutaneous involvement, and subcutaneous abscess. The language of publication, journal, or country was not included as limitation criteria, and publications dated before August 1, 2016, were considered. Articles or abstracts containing adequate information, such as age, sex, cyst size, cyst location, clinical presentation, fistula opening location, and management were included in the study, whereas articles with insufficient clinical and demographic data were excluded.

**Results:**

The literature review included 52 articles involving 55 patients with parietal complications of HD. Thirty-two articles were written in English, 15 in French, 2 in Spanish, 1 in Italian, 1 in German and 1 in Russian. All 55 patients (women, 30; men, 23; unknown, 2) involved in the study were aged 7 to 93 (mean ± standard deviation, 54.5 ± 20.2) years. A total of 24 patients had cysto-cutaneous fistula (*Echinococcus granulosus*), 12 had subcutaneous rupture, 10 had cutaneous fistula (*E multilocularis*), 3 had cystosubcutaneous abscess, 3 had cysto-cutaneo-bronchial fistula, 2 had cysto-cutaneo-bronchio-biliary fistula, and 1 had cutaneo-bronchial fistula. *E granulosus* were detected in 43 and *E multilocularis* in 12 patients through clinical, radiological, or histopathological examinations.

**Conclusion:**

Parietal complications such as cysto-cutaneous fistulization are a rare complication of HD. Complicated HD should be considered in the differential diagnosis of patients presenting with cutaneous involvement, especially in HD endemic regions.

## 1 Introduction

Hydatid disease (HD) is a zoonotic disorder caused by parasites that belong to the *Echinococcus* species of the Taeniidae family of cestodes. The *Echinococcus* species that most commonly causes HD in humans are *Echinococcus granulosus*, the causative agent of cystic echinococcosis, followed by *E multilocularis*, the causative agent of alveolar echinococcosis.^[1–54]^ Cystic echinococcosis (hydatid cyst) is responsible for 95% of all hydatid cyst cases in humans.^[2]^ Although HD may involve any tissues or organs in the human body, the liver (50%–77%), lungs (15%–47%), spleen (0.5%–8%), and kidneys (2%–4%) are the most commonly involved.^[2–8]^ After reaching a tissue or organ, hydatid cysts grow approximately 1 cm per year, explaining why most patients remain asymptomatic for years.^[6,23]^ Asymptomatic patients are usually diagnosed by radiological studies performed for other indications.^[12]^ However, a smaller proportion of patients may become symptomatic after the occurrence of complications, such as secondary infections, anaphylaxis, adjacent organ compression, and rupture.^[6,12]^ Secondary cyst infections occurring as a result of cysto-biliary communication are the most common risk factor for an increased intracystic pressure, erosion of adjacent structures by an enlarging cyst, and development of complications.^[6]^ Ruptures (perforation, fistulization) are the most severe complication. Hepatic hydatid cysts mainly rupture into the bile ducts, gastrointestinal tract, bronchi, peritoneal cavity, and pleural space, whereas pulmonary hydatid cysts mainly rupture into the pleural space, mediastinum, and bronchial tree.^[8]^ Subcutaneous progression followed by skin fistulization (rupture) of HD is a relatively uncommon complication,^[8,12]^ which has been reported in a limited number of cases to date. This study aimed to create awareness among readers by conducting a literature analysis to review studies on parietal complications of cutaneous and subcutaneous involvement of HD.

## 2 Materials and methods

### 2.1 Definition of the terms

A clear consensus is yet to be reached regarding the definition, complications, and treatment algorithm of HD. To summarize the correct terminology, all diseases caused by parasites of *Echinococcus* species are called HD. Diseases caused by *E granulosus*, *E multilocularis (alveolaris)*, *E vogeli*, and *E oligarthrus* are known as cystic echinococcosis (hydatid cyst), alveolar echinococcosis, polycystic echinococcosis, and unicystic echinococcosis, respectively. All complications that develop as a result of cutaneous communication of HD, involving organs and tissues in the thoracoabdominal cavities, are referred to as parietal complications,^[7,12,15]^ which are categorized into 3 stages depending on the depth of invasion of hydatid lesions on the abdominal or thoracic wall: stage 1, hydatid lesions protruding into the mural muscular layer; stage 2, those that pass beyond the muscular layer to invade or protrude into the subcutaneous layer; and stage 3, those that pass beyond the subcutaneous tissue to fistulize the skin, also known as external rupture or cutaneous fistulization.^[12]^ Our literature review was designed based on the previously mentioned staging system.

Cystic echinococcosis lesions are unilocular or multivesicular cystic lesions depending on the stage of a given cyst. Hence, cutaneous involvement by *E granulosus* is referred to as cysto-cutaneous fistula. Lesions of alveolar echinococcosis usually have a tumor-like, invading pattern. Therefore, skin fistula caused about by *E alveolaris* was referred to as cutaneous fistula.

The patients were categorized into spontaneous and postoperative rupture categories based on the pattern of occurrence and location of parietal complications. In conclusion, cases that had been operated for HD and developed a parietal complication from an incision scar or its surrounding tissues any time after surgery were referred to as postoperative rupture.^[3,5,11,15,24,32,40]^ Cases without a history of surgery and those that had a parietal complication at an anatomic location far from the scar tissue years after surgery were known as spontaneous rupture.^[5,8,13,16,22,29,34–36,46,52]^

### 2.2 Study design

A literature search was conducted on PubMed, Medline, Google Scholar, and Google databases in accordance with the Preferred Reporting Items for Systematic Reviews and Meta-Analyses guidelines using the following keywords.

HD, hydatid cyst, cystic echinococcosis, alveolar echinococcosis, abdominal HD, thoracic HD, parietal complication, cutaneous fistulization, cysto-cutaneous fistulization, cutaneous involvement, external rupture, external fistulization, subcutaneous involvement, and subcutaneous abscess alone or in different combinations (flow diagram). No language, journal name, and country restrictions were applied for the literature research. Google Translate was used for articles written in languages other than English and Turkish. A significant proportion of the scanned articles were written in French. The authors of these articles were e-mailed to get help in accurately analyzing their case reports. As no adequate support was obtained for proper translation of the articles, their introduction and discussion sections could not be used at all. No time window was determined for the study period, and all documents published on parietal complications of HD before August 1, 2016, were reviewed. The corresponding authors of the articles with a substantially large amount of missing information than that in other articles were e-mailed to obtain information on their cases.

As a result, articles without an accessible full-text version, those that did not provide adequate information in their abstracts, and those that did not involve as comprehensive information as that provided in other studies were excluded. As some enrolled articles were published in the form of a literature review, their tables were also used.^[5,12,23,45]^ The following information were collected: publication year, country, publication language, paper type (full-text, abstract, not available), age, sex, clinical presentation, type of *Echinococcus* spp. (*E granulosus*, *E alveolaris*), location of the fistula opening (topographically), cyst locations (liver, kidney, lung, etc), cyst size (mm), previous surgery, radiologic tools, neoadjuvant antiparasitic chemotherapy, surgical management, postoperative antiparasitic chemotherapy, recurrence, and follow-up (month). Because of the retrospective design of this literature review, we did not apply for ethics committee approval. Categorical variables were presented as number and percentage (%) and continuous variables, as mean ± standard deviation (SD).

## 3 Results

A total of 52 articles reporting 55 cases were deemed suitable for inclusion^[3–54]^: 42 full-text articles, 4 with abstract version only, and the remaining 6 articles that could not be accessed. Some missing information in 10 articles with no accessible full-text versions were obtained from other articles published as literature reviews.^[5,12,23,45]^ The top 7 countries of origin of the articles on parietal complications were India (n = 9), Tunisia (n = 6), Turkey (n = 5), France (n = 5), Morocco (n = 5), Italy (n = 4), and Spain (n = 4). A total of 32 articles were written in English, 15 in French, 2 in Spanish, and 1 each in German, Italian, and Russian.

The total number of patients was 55 (women, 30; men, 23; unknown, 2) aged 7 to 93 (mean ± SD, 54.4 ± 20.2) years, ranging from 29 to 87 (mean ± SD, 56.6 ± 16.5) years in men and 7 to 93 (mean ± SD, 53.1 ± 22.7) years in women. Both sexes showed no significant difference with respect to the mean age (*P* = .67). Other details are provided in Table 1  .

**Table 1:**
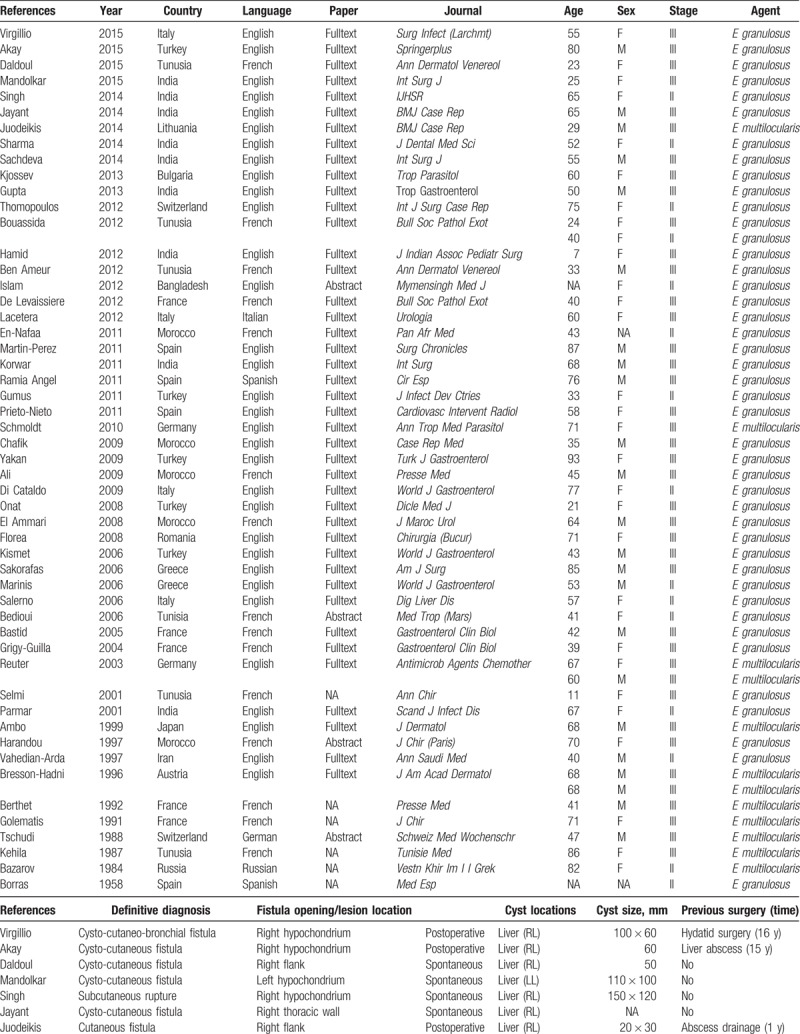
Demographic and clinical characteristics of 55 patients with parietal complication of the hydatid disease.

**Table 1 (Continued):**
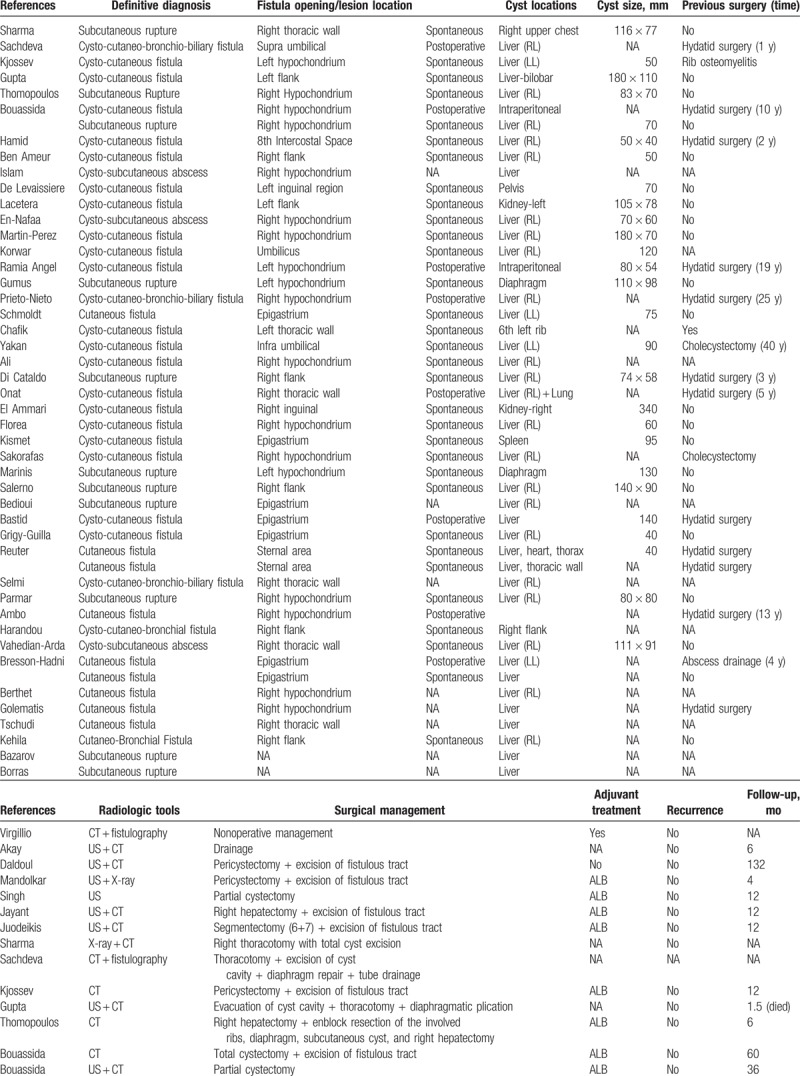
Demographic and clinical characteristics of 55 patients with parietal complication of the hydatid disease.

**Table 1 (Continued):**
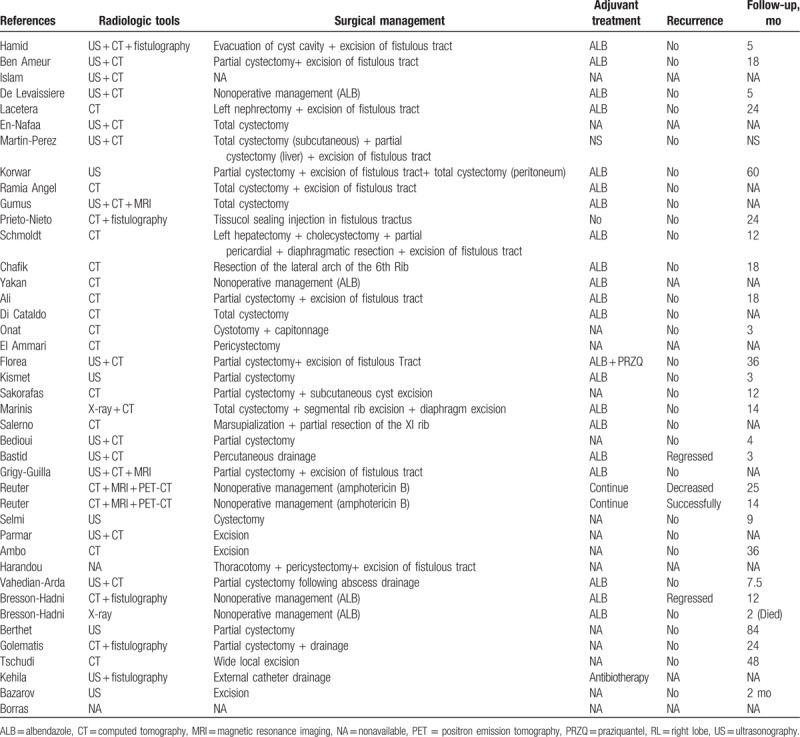
Demographic and clinical characteristics of 55 patients with parietal complication of the hydatid disease.

Twenty patients underwent abdominal or thoracic surgery before a parietal complication was diagnosed. Thirteen of them were operated for HD 1 to 25 years before the admission date (mean ± SD, 10.9 ± 7.7 years). Three patients underwent several drainage procedures for a preliminary diagnosis of intra-abdominal abscess. All of them had developed fistula after drainage, and the causative agents were *E multilocularis* (n = 2) and *E granulosus* (n = 1). A patient underwent multiple operations for a presumed fistulization secondary to rib osteomyelitis, although a hydatid cyst located in the left lobe of the liver fistulizing to the 11th rib had been the actual cause. Another patient had developed fracture and swelling of the left 6th rib after parachuting. He underwent drainage in the emergency department but continued to suffer a fistula thereafter. After receiving delayed diagnoses, both cases underwent appropriate surgical interventions and were administered with postoperative albendazole treatment. The remaining 2 patients underwent open cholecystectomy. Table 1   shows the patient's clinical characteristics.

Information on admission symptoms of 45 patients was obtained: 34 experienced the symptoms from 2 days to 25 years. The most common symptoms and signs were skin discharges and palpable mass/swelling. Nine patients were admitted solely for skin discharges, whereas 12 patients had at least one of the following signs and symptoms in addition to discharges: fever, abdominal pain, swelling, cough, and distension. Eleven patients presented to hospital only for a palpable mass/swelling, whereas 9 patients had at least one of the following signs and symptoms: abdominal pain, discharge, and fever. Six patients developed purulent skin discharge shortly after having swelling, fever, or distention. The case of right kidney hydatid cyst fistulizing to the skin presented with dysuria, pyuria, and membranous structures in the urine and was diagnosed after the cystic lesion had fistulized to the skin.

Twenty-three of 24 patients had a positive serological result, whereas 1 patient had a negative result. The results of preoperative radiological studies were accessed in 52 patients. Computed tomography (CT) had been used in 16 patients, ultrasonography (US) + CT in 16, US in 6, US + CT + magnetic resonance imaging (MRI) in 2, and CT + MRI + positron emission tomography in 2. A fistulography had been performed in addition to CT and/or US in 7 patients, of whom only 1 had a nondiagnostic fistulography result. Table 1   shows other details.

A subgroup analysis of parietal complications revealed that 24 patients had cysto-cutaneous fistula, 12 had subcutaneous rupture, 10 had cutaneous fistula, 3 had cysto-subcutaneous abscess, 3 had cysto-cutaneo-bronchial fistula, 2 had cysto-cutaneo-bronchio-biliary fistula, and 1 had cutaneo-bronchial fistula. A staging system based on the relationship between HD and the thoracoabdominal wall revealed that 40 and 15 patients had stage 3 (cutaneous involvement) and stage 2 (subcutaneous involvement) parietal complications, respectively. The etiological agent causing parietal complications were *E granulosus* in 43 patients and *E alveolaris* in 12 patients. Table 1   shows other details.

Information on the topographic location of parietal complications was obtained in 53 patients. Parietal complication occurred in the right hypochondrium in 17 patients, right flank in 7, epigastrium in 7, right thoracic wall in 6, left hypochondrium in 5, periumbilical region in 3, over the sternum in 2, left flank in 2, inguinal region in 2, 8th intercostal region in 1, left thoracic wall in 1, and unknown in 2.

Information on the pattern of occurrence of parietal complications was obtained in 47 patients. Spontaneous rupture occurred in 36 patients. Although 8 of these patients had been operated, the parietal complication and incision occurred in separate anatomic locations. A postoperative rupture was identified in 11 patients. All of these patients underwent surgery for HD (n = 8) or an abscess of unknown cause (n = 3).

Thirteen patients were administered with neoadjuvant anthelmintic therapy in the form of albendazole (n = 12) or albendazole + mebendazole (n = 1), whereas 2 patients with *E alveolaris* were administered long-term amphotericin B treatment, but no planned surgical procedure. Five patients did not receive neoadjuvant antihelminthic therapy. Whether the 35 patients were administered with preoperative antihelminthic therapy cannot be confirmed. Information on the neoadjuvant treatment protocol after surgery was obtained in 33 patients (albendazole = 26, albendazole + praziquantel = 1, benzimidazole = 1, antibiotherapy = 1, amphotericin B = 2, and none = 2), but unknown in 22 patients. Among the 55 patients, 7 did not undergo any surgical procedure, whereas 48 underwent at least 1 surgical procedure. However, as common terminology or treatment protocol was not available, we provided treatment modalities in Table 1   under the heading “Surgical Management.”

Thirty-seven patients were followed up for 40 days to 132 months, and only 2 of them reportedly died. One of the deceased patients had 3 cysts >10 cm originating from the liver and invading the diaphragm, pericardium, and abdominal wall. The patient suffered both respiratory difficulty and severe hypoalbuminemia and also needed inotropic support during surgery but eventually died 40 days postoperatively despite all efforts. Another patient who received medical treatment for *E alveolaris* died at the second month of treatment due to lung cancer in the left upper lobe, extensive emphysema, and infiltrative atelectasis. Information regarding the length of follow-up of 18 patients was not obtained.

A total of 43 patients did not develop any recurrence of parietal complications during follow-up; however, information on recurrence was not obtained in 8 patients. The remaining 4 patients experienced an improved fistula or regression of fistulous lesions. Two of these cases were previously diagnosed with *E alveolaris*. Both developed hepatotoxicity after benzimidazole treatment, and thus amphotericin B was administered as a novel treatment option. One of them achieved complete successful treatment, and the other experienced reduced lesion size and closed fistula. In a case with cutaneous fistula development due to *E multilocularis*, the progression of hepatic lesions was prevented, and nodular skin lesions significantly regressed with effective albendazole treatment. A patient was found to have 2 cystic hepatic lesions (8 and 14 cm in size) that had fistulized to the skin and stomach, respectively. The cysts were successfully treated and significantly reduced with percutaneous catheterization technique.

## 4 Discussion

HD is a significant public health problem in the Middle East, Far East, Mediterranean, South America, and Australia, where agriculture and animal husbandry are the primary means of living.^[4,8]^ Humans who have no role in the biological life cycle of the parasite are accidentally infected after ingesting eggs of *E granulosus* found in canine stool.^[8]^

As HD has a slow growth rate, a significant proportion of cases may remain asymptomatic for years. Hence, asymptomatic cases are incidentally diagnosed by radiological studies performed for other indications.^[6,8,12]^ However, a minority of cases may show some signs and symptoms depending on the size, number, and location of lesions; their relationship with the biliary tree and vascular structures; and compression of adjacent organs.^[4,8,34]^ The most common complications of hepatic HD are rupture (peritoneal, pleural, gastrointestinal, cutaneous), secondary infections (cyst abscess), anaphylactic reaction, and compression of vascular structures (Budd-Chiari syndrome, portal hypertension), biliary tree (cholangitis, obstructive jaundice), and adjacent organs (gastric outlet obstruction).^[8,16,29,34]^ Rupture of hepatic hydatid cysts into the bile ducts, gastrointestinal tract, bronchi, pleural space, and peritoneal cavity are referred to as internal rupture, the most common complication of HD.^[14,16]^ Despite being extremely rare, liver HD may penetrate the abdominal wall and the cysts may rupture outside the body, also known as external rupture (cutaneous fistulization).^[8]^

Increased intracystic pressure secondary to infection, erosion of adjacent walls (thoracic or abdominal) by cyst growth, and complications due to a cyst's migration toward the body surface are collectively known as parietal complications.^[23]^ A cyst's progression toward the thoracic and abdominal walls can be divided into 3 stages. Stage 1 (muscle) is characterized by cyst protrusion to the innermost layer of the wall, that is, peritoneal/pleural layer, and the layer immediately above it, that is, the muscular layer. Stage 2 (subcutaneous tissue) is characterized by passage beyond the muscular layer and protrusion into the subcutaneous soft tissue. Stage 3 (skin fistulization) is characterized by passage beyond the subcutaneous tissue and fistulization to the skin, also known as external rupture or cutaneous fistula,^[12]^ and it is a rare complication of HD.^[6]^ Cystic lesions caused by *E alveolaris* do not cause parietal complications alone. Instead, they infiltrate the skin by a tumor-like mechanism.

Another classification of hydatid cyst rupture was first published by Lewall and McCorkell ^[55]^ in 1986. The authors categorized hydatid cyst rupture into 3 categories: contained, communicating, and direct. Contained rupture occurs when only the endocyst layer ruptures and the cyst content is contained inside an intact pericyst cavity.^[12,55]^ Communicating refers to rupture of the endocyst layer and passage of the cyst content into the bronchioles or biliary tree.^[12,55]^ Direct rupture is characterized by the rupture of both endocyst and pericyst layers and the cyst content passes into adjacent organs and pleural and peritoneal cavities.^[12,55]^ It is the most complicated form of hepatic hydatid cysts and is associated with a greater risk than other categories.

The first step in diagnosing parietal complications is clinical suspicion and patient history: detecting a palpable lesion and external opening of a fistula on physical examination, a patient residing in rural areas or having contact with canines, or a surgical history of HD or having active HD. In patients presenting with cutaneous fistulization, a discharge of cyst fluid or daughter vesicles from the external opening of a fistula is the most common clinical sign. The histopathological analysis of the fluid drained through the external opening of a fistula may reveal multiple protoscoleces. Culture and antibiogram generation of cyst fluid can also be performed because superinfection exists in most complicated cysts.

The second step is the use of serological tests. The most commonly used tests both in diagnosing HD and monitoring for recurrence are enzyme-linked immunosorbent assay, indirect hemagglutination, serum immunoelectrophoresis, complement fixation test, and immunofluorescence assay. Among the 24 patients with accessible serological test results, 23 (95.8%) patients tested positive and 1 patient tested negative for *E granulosus*, but the presence of *E granulosus* was histopathologically confirmed.

The third step is the use of radiological instruments. The most commonly employed radiological tools both to diagnose parietal complications of HD and determine the location of lesions are as follows: US,^[7,17–19]^ CT,^[27–38]^ MRI,^[25,42]^ and fistulography.^[3,11,16,26,48,50,52]^ Fistulography may be help in determining the length of a fistula's internal orifice, the size and location of fistulized lesion, and the relationship of a fistula with bile ducts, bronchopleural structures, and pelvicalyceal system. A cutaneous fistulization was detected in 40 of the enrolled patients; 17.5% of whom were studied using fistulography in addition to other radiological instruments. Bresson-Hadni et al^[48]^ failed to show any connection between the fistula tract and hepatic lesion when they used fistulography. Prieto-Nieto et al^[26]^ used fistulography to identify the communication between the fistula tract and bronchobiliary structures in a patient who underwent remote hydatid cyst surgery. The authors reported that they successfully treated the patient by filling the fistula tract with Tissucol, a biological fibrin glue. In conclusion, the success rate of fistulography to determine communication between a fistula's external opening and an organ/cavity is 85.7%. In our opinion, fistulography is technically feasible and should be performed in all cases without any contraindication. Fistula extensions greatly help in determining the type of surgery. However, additional studies are needed to convey a clearer message to the reader regarding the use of fistulography in cases of cutaneous fistulization.

The most appropriate approach for the treatment of parietal complications is to perform an elective surgical intervention following neoadjuvant benzimidazole treatment for 2 to 4 weeks. The most appropriate surgical treatment for stage 1 and 2 parietal complications is total resection of protruded hydatid cysts, together with their extension from the organ of origin. The most appropriate surgical management for stage 3 parietal complications is en bloc resection of the primary hydatid cyst, fistula tract, and involved skin.^[6,8]^ Radical surgical approaches such as pericystectomy, segmentectomy, and lobectomy are the most suitable options to prevent recurrences, although they are associated with increased morbidity compared with conservative surgical approaches. Adjuvant therapy is unnecessary in cases scheduled for radical surgery. However, in complicated cases scheduled for conservative surgical methods such as partial cystectomy and cystotomy, the cyst cavity should be evacuated and reduced in size, and benzimidazole treatment should be administered for 4 to 12 weeks. Fascial defects formed in the abdominal or thoracic wall after resecting the skin and fistula tract are either closed primarily or cannot be possibly closed with artificial graft materials. In cases with superinfections or abscess formation, drainage should be made and specific antibiotics should be administered. Treatment of parietal complications with *E alveolaris* is quite more difficult and complicated than those with *E granulosus*. In the majority of cases, involvement of multiple cutaneous orifices or cutaneous areas may be identified. Furthermore, *E alveolaris* causes a tumor-like lesion in the primarily involved organ and in the abdominal wall.^[9]^ Cases of HD caused by *E alveolaris* cases that are suitable for resection should be treated by resecting both the primary lesion and the involved skin area, leaving a 1-cm surgical margin.^[9]^ No matter which surgical treatment option is used in cases with *E alveolaris*, administering long-term neoadjuvant and postoperative benzimidazole treatment is the most appropriate management approach to minimize the risk of recurrence.^[9]^ Approximately 60% of the patients presented in this literature review had been administered with adjuvant medical treatment for 1 to 12 months. Four of the treated patients diagnosed with extensive *E alveolaris* were administered with long-term medical treatment without undergoing surgical procedures. In this review, the most interesting study is that by Reuter et al,^[42]^ who achieved a successful outcome with amphotericin B in 2 patients with hepatic toxicity after benzimidazole treatment. To our knowledge, only 4 studies reported the use of amphotericin B for the treatment of patients with *E alveolaris*, 3 of which have been reported by Reuter et al. The authors recommended the use of amphotericin B for benzimidazole-intolerant or treatment-resistant cases.^[42]^

In conclusion, as the number of cases reporting cutaneous fistulization of HD is limited, we cannot make robust recommendations regarding its management. Indeed, this subject should be further investigated by further studies.

### 4.1 Topic highlight

Cutaneous fistulization is a rare but serious complication of HD. Cases without cyst fluid or daughter vesicle discharges from the external opening of a fistula cannot be easily diagnosed. One of the objectives of the present study is to raise awareness of the disease among physicians working in endemic regions.

Many terms have been used in the literature to define the relationship between HD and thoracoabdominal wall, such as cutaneous fistulization, cysto-cutaneous fistulization, external rupture, external fistulization, and skin rupture. All of these terms should be ideally considered under the title “parietal complications” to form an accurate and common terminology.

Although a consensus is yet to be reached regarding the parietal complications of HD, the general principles in treating HD may also be used in these cases. The most appropriate approach is to perform a radical resection involving the fistula tract (if possible) after neoadjuvant therapy and to administer long-term adjuvant medical treatment, particularly for alveolar echinococcosis.

## Author contributions

Akbulut S designed the literature review, organized the report, and wrote the paper.

**Conceptualization:** Sami Akbulut.

**Data curation:** Sami Akbulut.

**Formal analysis:** Sami Akbulut.

**Project administration:** Sami Akbulut.

**Supervision:** Sami Akbulut.

**Visualization:** Sami Akbulut.

**Writing – original draft:** Sami Akbulut.

**Writing – review and editing:** Sami Akbulut.
